# Conjunctival Mucosa-Associated Lymphoid Tissue (MALT) Lymphoma Associated With Methotrexate Therapy: A Case of Complete Remission Following Drug Discontinuation

**DOI:** 10.7759/cureus.91887

**Published:** 2025-09-09

**Authors:** Kazuya Shimada, Hideki Fukuoka, Minori Minamide, Toshihide Ikeda, Chie Sotozono

**Affiliations:** 1 Department of Ophthalmology, Kyoto Prefectural University of Medicine, Kyoto, JPN

**Keywords:** conjunctiva, lymphoproliferative disorder, malt lymphoma, methotrexate, rheumatoid arthritis

## Abstract

Methotrexate-associated lymphoproliferative disorders (MTX-LPDs) are a distinct clinical entity with potential for spontaneous regression after drug discontinuation. We report the case of a 64-year-old man with rheumatoid arthritis treated with MTX 4 mg twice weekly (8 mg/week) for 11 years, who developed a salmon-pink, lobulated conjunctival mass in the left superior nasal quadrant. Laboratory evaluation showed elevated immunoglobulins, the presence of M-protein, and serum Epstein-Barr virus (EBV)-DNA of 2.18 U/mL. Biopsy confirmed mucosa-associated lymphoid tissue (MALT) lymphoma. MTX was promptly discontinued, and the mass was excised. At 10 months post-withdrawal, the patient achieved complete clinical and biochemical remission, with the disappearance of M-protein and no evidence of recurrence. This case underscores the importance of recognizing MTX-LPD in patients receiving MTX, as prompt drug discontinuation can lead to complete regression without chemotherapy. The conjunctival presentation is rare and broadens the recognized anatomical spectrum of MTX-LPD.

## Introduction

Methotrexate (MTX) remains the primary treatment for rheumatoid arthritis; however, its widespread use has led to increasing recognition of MTX-associated lymphoproliferative disorders (MTX-LPDs). These disorders are a distinct subset of iatrogenic immunodeficiency-associated lymphoproliferative disorders, notable for their potential to regress spontaneously after MTX discontinuation [1,2].

MTX-LPDs typically present with lymphadenopathy but often involve extranodal sites such as the lungs, oral cavity, nasopharynx, and skin [1]. Their pathogenesis is linked to MTX-induced immunosuppression, which can trigger Epstein-Barr virus (EBV) reactivation in about 60%-75% of cases [2,3]. A key feature of MTX-LPDs is their ability to regress spontaneously after MTX withdrawal, observed in 60%-75% of cases. Regression is more frequent in EBV-positive cases (85.2%) compared to EBV-negative cases (50%) [1,2].

The occurrence of MTX-associated MALT lymphoma presenting as a conjunctival lesion is extremely rare, with only a few cases reported in the literature [4]. Recognizing this entity is important, as prompt MTX discontinuation may prevent the need for aggressive treatment while still achieving favorable outcomes. This case report aims to raise clinical awareness of this underrecognized but treatable complication of MTX therapy.

## Case presentation

A 64-year-old man with a 15-year history of rheumatoid arthritis presented with a six-month history of progressive swelling in the left conjunctiva. His rheumatoid arthritis had been managed with MTX 4 mg twice weekly (8 mg/week) for 11 years, along with etanercept 50 mg injections initiated one year earlier due to disease flares. Additional medications included folic acid supplementation, celecoxib 100 mg daily, and proton pump inhibitors for a history of peptic ulcer disease (*Helicobacter pylori* eradicated).

**Figure 1:**
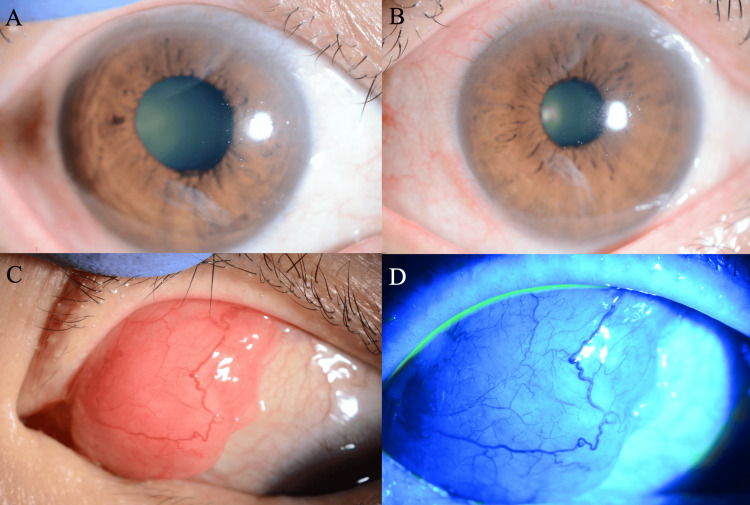
Clinical presentation at initial examination (A) Anterior segment photography of the right eye showing normal conjunctival appearance with no abnormal findings. (B) Anterior segment photography of the left eye in primary gaze demonstrating mild conjunctival injection and subtle tissue thickening, with the conjunctival mass not clearly visible from this angle. (C) Left eye with temporal gaze revealing the prominent salmon-pink conjunctival mass located in the superior nasal quadrant. The lesion demonstrates the characteristic smooth, lobulated appearance with well-defined borders and associated mild eyelid swelling. (D) Fluorescein staining of the left eye confirming no conjunctival ulceration. The intact surface epithelium overlies the underlying conjunctival lesion.

On physical examination, a prominent salmon-pink conjunctival mass was observed in the left eye, accompanied by mild eyelid swelling (Figure 1).

Corrected visual acuity was preserved (both eyes 20/20), and intraocular pressures were within normal limits (right 14.7 mmHg, left 18.3 mmHg). Systemic examination demonstrated left submandibular and cervical lymphadenopathy.

Laboratory investigations revealed significantly elevated immunoglobulins: IgG, 2,314 mg/dL (normal: 861-1,747 mg/dL); IgA, 1,826 mg/dL (normal: 93-393 mg/dL); and IgM, 121 mg/dL (normal: 33-183 mg/dL). Complete blood count showed the following: WBC 4,000/μL, RBC 456 × 10⁴/μL, Hb 13.4 g/dL, Hct 41.3%, MCV 90.6 fL, and PLT 14.8×10⁴/μL, all within normal limits. Peripheral blood smear examination revealed no circulating abnormal lymphocytes or cytopenias, ruling out systemic lymphomatous involvement. Serum protein electrophoresis demonstrated a prominent M-protein peak in the gamma region (23.9%, elevated), with a reduced albumin/globulin ratio of 0.7. Additional findings included elevated serum EBV-DNA at 2.18 U/mL (normal < 1.5 U/mL), soluble interleukin-2 receptor of 431 U/mL (normal: 157-474 U/mL), and elevated beta-2 microglobulin of 2.75 mg/L (normal: 0.8-2.0 mg/L). EBV serology revealed elevated IgG antibodies with normal IgM levels, confirming prior EBV exposure and indicating viral reactivation rather than primary infection.

Computed tomography imaging revealed thymic hyperplasia and generalized small lymphadenopathy throughout the neck to pelvis, consistent with systemic lymphoproliferative disease.

Surgical excision of the left conjunctival tumor was performed (Video 1).

**Video 1:** Performed surgical excision of the left conjunctival tumor

**Figure 2:**
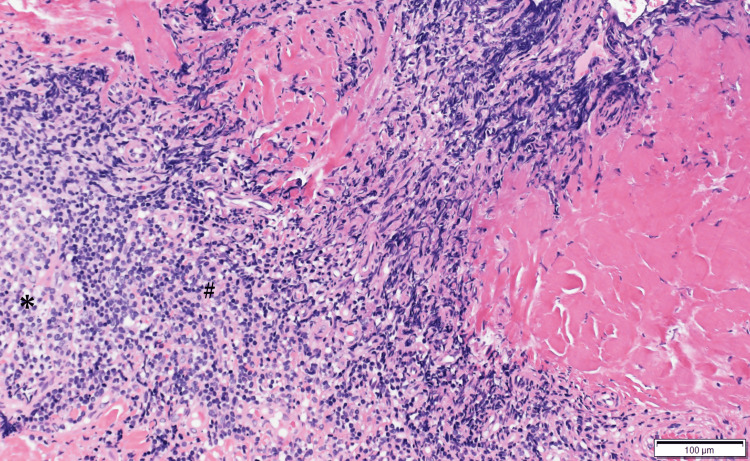
Histopathological examination of the conjunctival lesion Hematoxylin and eosin staining of the excised conjunctival tissue showing characteristic features of extranodal marginal zone B-cell lymphoma of mucosa-associated lymphoid tissue (MALT) type. The tissue demonstrates dense lymphoid infiltrate in the subepithelial stroma with formation of reactive follicles (asterisk, *). Small to medium-sized lymphocytes and plasma cells show monotonous proliferation around the follicles with follicular colonization (hash mark, #). The lymphoid cells exhibit characteristic centrocyte-like morphology typical of MALT lymphoma. Immunohistochemical analysis revealed monoclonal light chain restriction with kappa predominance over lambda (kappa >> lambda), confirming the diagnosis of MALT lymphoma. Scale bar = 100 μm.

Histopathological examination confirmed extranodal marginal zone B-cell lymphoma of MALT type (Figure 2).

Notably, EBV-encoded RNA (EBER) in situ hybridization performed on the excised tissue was negative, despite the elevated serum EBV-DNA at presentation. Bone marrow biopsy performed several weeks later showed no evidence of malignant involvement.

Based on the clinical presentation, laboratory findings, and histological confirmation, a diagnosis of MTX-associated conjunctival MALT lymphoma was established. MTX was immediately discontinued upon initial suspicion of MTX-LPD, and the patient was managed with watchful waiting. Rheumatoid arthritis treatment was continued with etanercept monotherapy, which the patient had been receiving for one year prior to MTX discontinuation.

**Figure 3:**
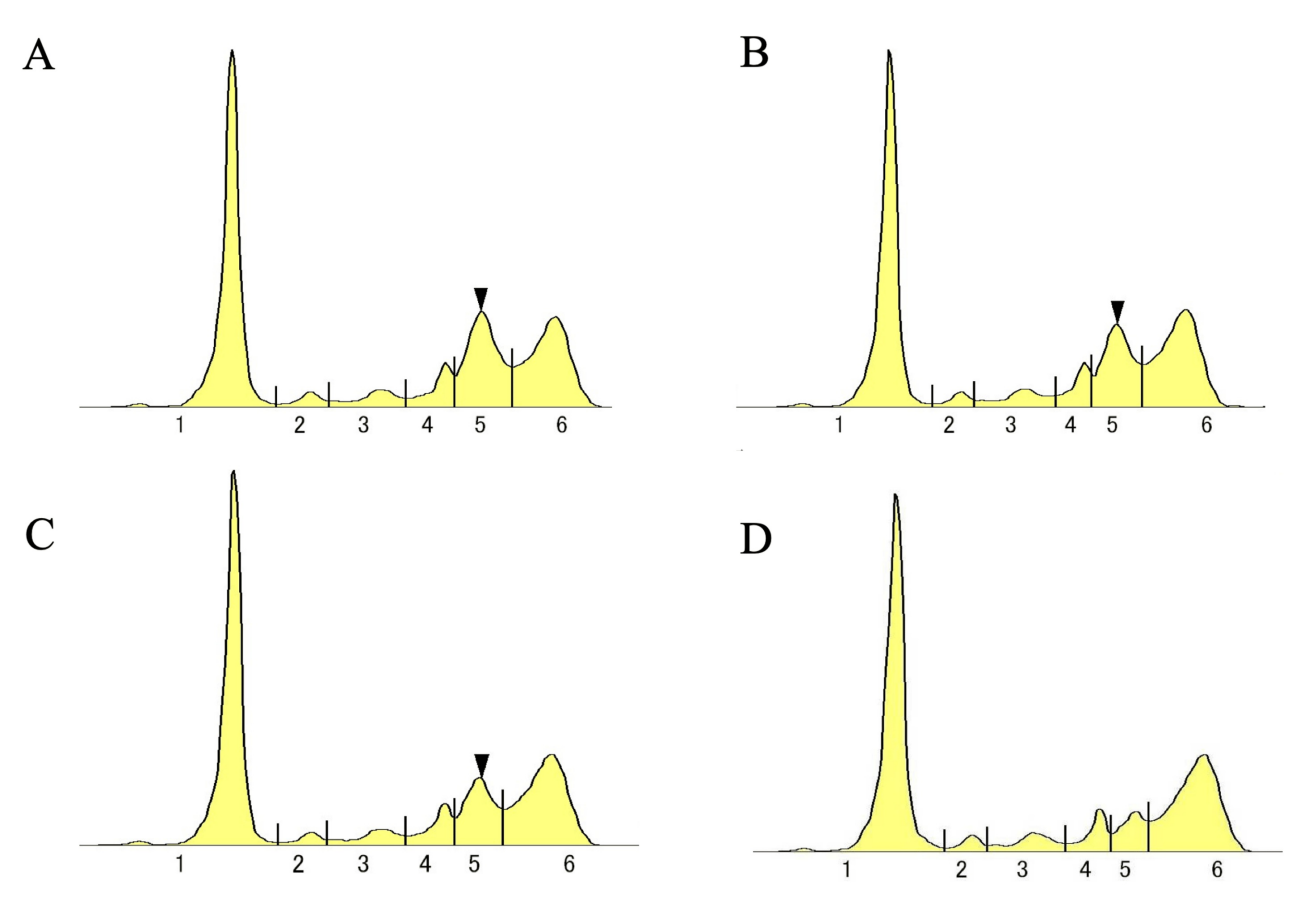
Serial serum protein electrophoresis demonstrating progressive M-protein resolution following methotrexate discontinuation Serial serum protein electrophoresis patterns showing the gradual disappearance of the M-protein peak (arrowheads) in the gamma region following methotrexate (MTX) withdrawal. (A) At MTX discontinuation (0 months): prominent M-protein peak with beta-2 globulin fraction of 20.5%. (B) Four months post-MTX discontinuation: reduced M-protein peak with beta-2 globulin fraction of 17.0%. (C) Six months post-MTX discontinuation: further reduction in M-protein peak with beta-2 globulin fraction of 14.1%. (D) Ten months post-MTX discontinuation: complete disappearance of M-protein peak with beta-2 globulin fraction normalized to 7.1%, indicating complete biochemical remission. Numbers 1-6 in the serum protein electrophoresis figure indicate the standard protein fractions: (1) albumin, (2) α1-globulin, (3) α2-globulin, (4) β-globulin, (5) γ-globulin, and (6) late γ-globulin.

The clinical course following MTX discontinuation was remarkable. Serial serum protein electrophoresis demonstrated progressive reduction in the M-protein peak, with complete disappearance achieved at 10 months post-MTX withdrawal. The conjunctival tumor excision site showed no evidence of local recurrence, and systemic lymphadenopathy gradually resolved. The patient remained clinically asymptomatic throughout the observation period (Figure 3).

**Figure 4:**
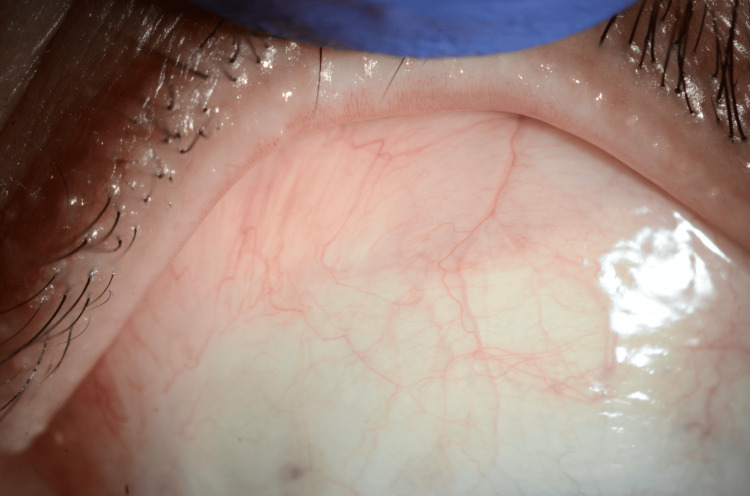
Complete resolution of conjunctival mucosa-associated lymphoid tissue (MALT) lymphoma one year following methotrexate discontinuation Clinical photograph of the left conjunctiva one year after surgical excision and methotrexate withdrawal showing complete healing of the excision site with no evidence of tumor recurrence.

At the most recent follow-up, the patient continues in complete clinical and biochemical remission with no evidence of lymphoma recurrence (Figure 4).

Rheumatoid arthritis management was successfully continued with etanercept monotherapy following MTX discontinuation. The patient maintained excellent disease control with no joint symptoms or disease flares throughout the follow-up period, demonstrating that effective rheumatoid arthritis treatment could be maintained without MTX.

## Discussion

This case exemplifies several critical educational points about MTX-associated MALT lymphoma that warrant emphasis for practicing clinicians. The complete regression following MTX discontinuation represents the most significant feature distinguishing MTX-LPDs from conventional lymphomas and fundamentally alters the therapeutic approach [1,2].

The presentation of a conjunctival mass in a long-term MTX user should immediately raise suspicion for MTX-LPD, particularly given the characteristic laboratory pattern of hypergammaglobulinemia with M-protein and elevated serum EBV-DNA. The conjunctival location is exceptionally rare for MTX-LPDs [4], expanding the recognized anatomical spectrum beyond the more commonly reported pulmonary, cutaneous, and oral cavity presentations [1].

The elevated serum EBV-DNA (2.18 U/mL) in this case supports the established pathogenic role of EBV reactivation in MTX-LPDs [3]. The serological pattern (IgG positive, IgM negative) definitively confirmed that this represented EBV reactivation in the setting of MTX-induced immunosuppression rather than primary viral infection. Interestingly, despite positive serum EBV-DNA at presentation, EBER in situ hybridization on the excised tissue was negative, likely reflecting rapid EBV clearance following immune function recovery after MTX discontinuation. This discordance between serum and tissue EBV status has been reported in MTX-LPDs and does not preclude the diagnosis [5]. The positive serum EBV-DNA finding, combined with the characteristic M-protein pattern, provides strong supporting evidence for the MTX-LPD diagnosis even before tissue confirmation.

The immediate MTX discontinuation upon clinical suspicion represents optimal management and should not be delayed pending tissue diagnosis [2]. This case demonstrates the excellent response potential of EBV-positive MTX-LPDs, with complete M-protein disappearance at 10 months, consistent with published regression rates of 85.2% for EBV-positive cases [1].

The serial monitoring of serum protein electrophoresis provides an excellent biomarker for treatment response, offering a non-invasive method to assess regression progress [6,7]. The 10-month timeframe to complete M-protein resolution aligns with literature showing maximum response typically occurring within 2-24 months of MTX cessation [6].

This patient exhibited several established risk factors for MTX-LPD development, including advanced age (64 years), prolonged MTX exposure (11 years), and concurrent immunosuppressive therapy with etanercept [2,8]. The MTX dose of 8 mg weekly was within the standard therapeutic range but still carried risk for MTX-LPD development, particularly given the prolonged exposure duration of 11 years, highlighting the importance of continued vigilance even with standard dosing [2].

The excellent long-term prognosis following successful regression must be balanced against the documented relapse rate in MTX-LPD patients [9,10]. This necessitates continued surveillance with regular clinical examinations and monitoring of serum markers. The successful transition to alternative rheumatoid arthritis therapies demonstrates that effective disease control can be maintained without MTX.

This case reinforces the need for high clinical suspicion in any patient on long-term MTX presenting with extranodal masses, lymphadenopathy, or laboratory abnormalities suggestive of lymphoproliferative disease [1,2]. The multidisciplinary approach involving rheumatology, ophthalmology, hematology/oncology, and pathology proved essential for optimal management.

The conjunctival presentation of MTX-associated MALT lymphoma represents a previously underrecognized manifestation that expands our understanding of the anatomical spectrum of these disorders [3]. This case represents one of the few reported instances of conjunctival MTX-associated MALT lymphoma and emphasizes the need for ophthalmologists to be aware of this rare but potentially reversible complication in MTX-treated patients.

## Conclusions

This case demonstrates the critical importance of recognizing MTX-associated MALT lymphoma as a potentially reversible complication of MTX therapy. Immediate MTX discontinuation should be the first-line intervention upon suspicion, with the potential for complete regression without chemotherapy. The conjunctival presentation expands the recognized anatomical spectrum of MTX-LPDs and emphasizes the need for heightened awareness among ophthalmologists and other specialists caring for MTX-treated patients.
